# The influence of stage at diagnosis and molecular subtype on breast cancer patient survival: a hospital-based multi-center study

**DOI:** 10.1186/s40880-017-0250-3

**Published:** 2017-10-25

**Authors:** Tingting Zuo, Hongmei Zeng, Huichao Li, Shuo Liu, Lei Yang, Changfa Xia, Rongshou Zheng, Fei Ma, Lifang Liu, Ning Wang, Lixue Xuan, Wanqing Chen

**Affiliations:** 1 National Office for Cancer Prevention and Control & National Central Cancer Registry, National Cancer Center/Cancer Hospital, Chinese Academy of Medical Sciences and Peking Union Medical College, Beijing, 100021 P. R. China; 2 Key Laboratory of Carcinogenesis and Translation Research (Ministry of Education/Beijing), Beijing Office for Cancer Prevention and Control, Peking University Cancer Hospital & Institute, Beijing, 100142 P. R. China; 3 Department of Medical Oncology, National Cancer Center/Cancer Hospital, Chinese Academy of Medical Sciences and Peking Union Medical College, Beijing, 100021 P. R. China; 4 Department of Statistics, The European Organization for Research and Treatment of Cancer (EORTC), 111200 Brussels, Belgium; 5 Department of Breast Surgery, National Cancer Center/Cancer Hospital, Chinese Academy of Medical Sciences and Peking Union Medical College, Beijing, 100021 P. R. China

**Keywords:** Breast cancer, Stage, Molecular subtype, Survival, China

## Abstract

**Background:**

Stage at diagnosis and molecular subtype are important clinical factors associated with breast cancer patient survival. However, subgroup survival data from a large study sample are limited in China. To estimate the survival differences among patients with different stages and various subtypes of breast cancer, we conducted a hospital-based multi-center study on breast cancer in Beijing, China.

**Methods:**

All resident patients diagnosed with primary, invasive breast cancer between January 1, 2006 and December 31, 2010 from four selected hospitals in Beijing were included and followed up until December 31, 2015. Hospital-based data of stage at diagnosis, hormone receptor status, and selected clinical characteristics, including body mass index (BMI), menopausal status, histological grade, and histological type, were collected from the medical records of the study subjects. Overall survival (OS) and cancer-specific survival (CSS) were estimated. Cox proportional hazards models were employed to evaluate the associations of stage at diagnosis and molecular subtype with patient survival.

**Results:**

The 5-year OS and CSS rates for all patients were 89.4% and 90.3%. Survival varied by stage and molecular subtype. The 5-year OS rates for patients with stage I, II, III, and IV diseases were 96.5%, 91.6%, 74.8%, and 40.7%, respectively, and the corresponding estimates of 5-year CSS rates were 97.1%, 92.6%, 75.6%, and 42.7%, respectively. The 5-year OS rates for patients with luminal A, luminal B, HER2, and triple-negative subtypes of breast cancer were 92.6%, 88.4%, 83.6%, and 82.9%, respectively, and the corresponding estimates of 5-year CSS rates were 93.2%, 89.1%, 85.4%, and 83.5%, respectively. Multivariate analysis showed that stage at diagnosis and molecular subtype were important prognostic factors for breast cancer.

**Conclusions:**

Survival of breast cancer patients varied significantly by stage and molecular subtype. Cancer screening is encouraged for the early detection and early diagnosis of breast cancer. More advanced therapies and health care policies are needed on HER2 and triple-negative subtypes.

## Background

Breast cancer is the most common cancer in women of China, accounting for 15% of all new cancers in women in 2015 [1], and women in urban areas had higher incidences than those in rural areas [2]. In Beijing, the capital and one of the largest cities of China, new breast cancer cases accounted for 22% of all new cancer cases in women between 2008 and 2012 [3]. In recent years, partly due to the changes in reproduction factors (e.g., nulliparity, mean number of children, age at first birth, and breastfeeding), the increasing use of hormone therapy and oral contraceptives, and more westernized dietary habits [4, 5], there were increasing trends of breast cancer incidence and mortality in China [1, 6].

Patient survival is a valuable medical indicator in evaluating the effectiveness and progress of breast cancer control. Stage at diagnosis is a key prognostic factor for breast cancer [7]. The disparities in breast cancer patient survival between Europe and the United States (US) were mainly explained by the lower proportion of advanced breast cancers in the US than in Europe [8]. Furthermore, breast cancer is a heterogeneous disease with distinct biological features, clinical behaviors, treatment responses, and outcomes according to its biological subtypes [9, 10]. However, in-depth analyses on survival have often been single-center studies with limited representativeness of the population [11, 12]. In China, few studies based on cancer registration data reported survival by different molecular subtypes of breast cancer [13].

In the present study, we conducted a multi-center, hospital-based study on breast cancer patient survival in Beijing, China. Meanwhile, using both active and passive follow-up systems of the population-based Beijing cancer registry, we were able to retrieve the information on the exact vital status of the study patients. We systematically analyzed breast cancer patient survival combining information of stage and molecular subtype with a large sample size, which will help us know the influence of related factors on breast cancer patient survival better.

## Methods

### Study population

All the Beijing residents who were newly diagnosed with breast cancer between January 1, 2006 and December 31, 2010 at four well-established hospitals were selected. The four involved hospitals were Cancer Hospital/Chinese Academy of Medical Sciences, Peking University Cancer Hospital, Beijing Obstetrics and Gynecology Hospital, and Shunyi Maternal and Child Health Care Hospital. Three of them were tertiary-level facilities, and one was secondary-level facility. Tertiary-level hospitals refer to the facilities that provide high-level medical and health care services to several districts with more hospital beds. Secondary-level hospitals have relatively less hospital beds and provide health care services to several communities. All female patients with primary invasive breast cancer were included in the final analysis. Exclusion criteria were as follows: male patients, non-invasive cancer, in situ cancer, or unknown vital status.

### Clinical data collection

Personal and clinical information on name, address, age, date of diagnosis, height, weight, menopausal status, histological grade, histological type, stage at diagnosis, estrogen receptor (ER) status, progesterone receptor (PR) status, human epidermal growth factor receptor 2 (HER2) status, and fluorescence in situ hybridization (FISH) results were abstracted from the medical records archived at the involved hospitals by trained investigators. Cancer staging was recorded according to the 7th edition of the American Joint Committee on Cancer (AJCC) criteria [14]. Molecular subtype was classified as luminal A (ER+ and/or PR+, HER2−), luminal B (ER+ and/or PR+, HER2+), HER2 (ER−, PR−, HER2+), triple-negative (ER−, PR−, HER2−) [15]. We defined HER2(–) or HER2(+) as negative expression, HER2(2+) as borderline expression, and HER2(3+) as positive expression. The cases with HER2 borderline expression would be further classified with FISH test.

### Follow-up

The detailed follow-up data of selected patients were collected from the database of Beijing cancer registry. The registry archives all newly diagnosed cancer patients’ records from all levels of clinics and hospitals, health insurance databases, death surveillance databases, the database of the basic medical insurances for urban residents, and the new-rural cooperative medical system. The registry routinely uses both active and passive follow-up methods to identify the survival statuses of cancer patients since the date of diagnosis. Passive follow-up was conducted by linking cancer registration records to the Beijing vital statistical database. The exact cause of death would be recorded in the death certification report when the patient died. Once the patient’s personal identification record did not match with the Beijing vital statistical database, the registry would use active follow-up methods including home visits or telephone contact to retrieve his/her exact survival information and cause of deaths. All the patients were followed up until December 31, 2015.

### Statistical analysis

Overall survival (OS) was defined as the time from diagnosis to death from any cause. Cancer-specific survival (CSS) was the time from diagnosis to death due to breast cancer. Data were censored if no endpoint event was observed during the study period. OS and CSS were estimated with the life-table method using the log-rank test for the detection of observed differences. Associations of breast cancer stage and molecular subtype with clinicopathologic features were examined using one-way analysis of variance (ANOVA) tests for continuous variables, and Chi square or Fisher exact test was used for categorical variables. Multivariable Cox proportional hazards models were employed to evaluate the associations between key prognostic factors, including stage at diagnosis, molecular subtype, and other mentioned factors, and survival outcome. Hazard ratios (HRs) were adjusted for age, body mass index (BMI), menopausal status, histological grade, histological type, cancer stage, and molecular subtype. All statistical analyses were conducted using SAS software version 9.2 (SAS Institute Inc, Cary, NC, USA).

## Results

### Demographic and clinical characteristics of patients

A total of 5044 patients diagnosed between 2006 and 2010 were identified, covering about 40.9% of all newly diagnosed breast cancer cases reported to the Beijing cancer registry. Detailed clinical information of these patients was collected. Among these patients, 404 were excluded for the following reasons: male (*n* = 16), non-invasive or in situ breast cancer (*n* = 135), unknown vital status (*n* = 249), and unknown cause of death (*n* = 4). A total of 4640 female patients with primary, invasive breast cancer were selected.

BMI (*P* < 0.01), menopausal status (*P* = 0.03), histological grade (*P* < 0.01), and molecular subtype (*P* < 0.01) showed significant differences by stage at diagnosis (Table 1). Patients with late-stage (stage III and stage IV) tumors were more likely to be triple-negative, at postmenopausal status, and with high BMI than those with early-stage (stage I and stage II) tumors.

**Table 1:** Characteristics of breast cancer patients by stage at diagnosis

Characteristic	Overall	Stage					*P* value^b^
		I	II	III	IV	Unknown^a^	
Total	4640	1334	2180	573	100	453	
Age (years)	52.9 ± 11.3	53.1 ± 11.5	52.9 ± 11.3	52.6 ± 11.0	53.3 ± 11.0	55.7 ± 13.6	0.85
BMI	25.0 ± 3.8	24.5 ± 3.6	25.2 ± 3.9	25.5 ± 4.0	25.0 ± 3.5	25.2 ± 3.9	< 0.01
Menopausal status							0.03
Premenopausal	2017 (43.5)	616 (46.2)	956 (43.9)	244 (42.6)	36 (36.0)	165 (36.4)	
Postmenopausal	2510 (54.1)	691 (51.8)	1175 (53.9)	318 (55.5)	62 (62.0)	264 (58.3)	
Unknown^a^	113 (2.4)	27 (2.0)	49 (2.2)	11 (1.9)	2 (2.0)	24 (5.3)	
Histological grade							< 0.01
Well differentiated	349 (7.5)	147 (11.0)	136 (6.2)	20 (3.5)	5 (5.0)	41 (9.1)	
Moderately differentiated	2549 (54.9)	714 (53.5)	1257 (57.7)	311 (54.3)	48 (48.0)	219 (48.3)	
Poorly differentiated	899 (19.4)	224 (16.8)	461 (21.1)	140 (24.4)	15 (15.0)	59 (13.0)	
Unknown^a^	843 (18.2)	249 (18.7)	326 (15.0)	102 (17.8)	32 (32.0)	134 (29.6)	
Histological type							0.28
Ductal	4031 (86.9)	1161 (87.0)	1928 (88.4)	509 (88.8)	88 (88.0)	345 (76.2)	
Lobular	149 (3.2)	43 (3.2)	60 (2.8)	23 (4.0)	3 (3.0)	20 (4.4)	
Others	460 (9.9)	130 (9.8)	192 (8.8)	41 (7.2)	9 (9.0)	88 (19.4)	
Molecular subtype							< 0.01
Luminal A	2387 (51.4)	775 (58.1)	1122 (51.5)	267 (46.6)	32 (32.0)	191 (42.2)	
Luminal B	535 (11.5)	144 (10.8)	246 (11.3)	72 (12.6)	18 (18.0)	55 (12.1)	
HER2	365 (8.0)	78 (5.9)	178 (8.2)	67 (11.7)	11 (11.0)	31 (6.9)	
Triple-negative	517 (11.1)	130 (9.7)	241 (11.1)	81 (14.1)	10 (10.0)	55 (12.1)	
Unknown^a^	836 (18.0)	207 (15.5)	393 (18.0)	86 (15.0)	29 (29.0)	121 (26.7)	

Continuous data are expressed as mean ± standard deviation (SD). Categorical data are expressed as number of cases (percent)

^a^Unknown data were not included in the statistical tests

^b^ *P* values were calculated using one-way analysis of variance (ANOVA) test (continuous variables) and Chi square or exact test (categorical variables)

Age (*P* = 0.01), menopausal status (*P* < 0.01), stage (*P* < 0.01), histological grade (*P* < 0.01), and histological type (*P* < 0.01) showed significant differences by molecular subtype (Table 2). The average age of patients with luminal A subtype of breast cancer was elder than the patients with other subtypes (*P* = 0.01). Patients with HER2 subtype were more likely to be postmenopausal, at late stage (III and IV), and had ductal breast cancer than patients with other subtypes (all *P* < 0.01). Patients with triple-negative tumors tended to have poorly differentiated tumor and at advanced stage (III and IV) compared with patients with luminal A and luminal B subtypes (all *P* < 0.01).

**Table 2:** Characteristics of breast cancer patients by molecular subtype

Characteristic	Overall	Subtype					*P* value^b^
		Luminal A	Luminal B	HER2	Triple-negative	Unknown^a^	
Total	4640	2387	535	365	517	836	
Age (years)	52.9 ± 11.3	53.5 ± 11.8	51.7 ± 10.6	52.9 ± 10.1	53.3 ± 11.8	53.2 ± 11.8	0.01
BMI	25.0 ± 3.8	25.1 ± 3.8	24.6 ± 3.8	25.3 ± 4.1	24.9 ± 3.7	25.1 ± 3.8	0.07
Menopausal status							< 0.01
Premenopausal	2017 (43.5)	1085 (45.5)	241 (45.1)	129 (35.3)	205 (39.7)	357 (42.7)	
Postmenopausal	2510 (54.1)	1251 (52.4)	280 (52.3)	232 (63.6)	303 (58.6)	444 (53.1)	
Unknown^a^	113 (2.4)	51 (2.1)	14 (2.6)	4 (1.1)	9 (1.7)	35 (4.2)	
Histological grade							< 0.01
Well differentiated	349 (7.5)	227 (9.5)	24 (4.5)	12 (3.3)	18 (3.5)	68 (8.1)	
Moderately differentiated	2549 (54.9)	1414 (59.2)	319 (59.6)	180 (49.3)	207 (40.0)	429 (51.3)	
Poorly differentiated	899 (19.4)	306 (12.8)	131 (24.5)	124 (34.0)	193 (37.3)	145 (17.4)	
Unknown^a^	843 (18.2)	440 (18.4)	61 (11.4)	49 (13.4)	99 (19.2)	194 (23.2)	
Histological type							< 0.01
Ductal	4031 (86.9)	2052 (86.0)	496 (92.7)	338 (92.6)	445 (86.1)	700 (83.7)	
Lobular	149 (3.2)	100 (4.2)	8 (1.5)	3 (0.8)	9 (1.7)	29 (3.5)	
Others	460 (9.9)	235 (9.8)	31 (5.8)	24 (6.6)	63 (12.2)	107 (12.8)	
Stage							< 0.01
I	1334 (28.7)	775 (32.5)	144 (26.9)	78 (21.4)	130 (25.2)	207 (24.7)	
II	2180 (47.0)	1122 (47.0)	246 (46.0)	178 (48.8)	241 (46.6)	393 (47.0)	
III	573 (12.3)	267 (11.2)	72 (13.5)	67 (18.4)	81 (15.7)	86 (10.3)	
IV	100 (2.2)	32 (1.3)	18 (3.4)	11 (3.0)	10 (1.9)	29 (3.5)	
Unknown^a^	453 (9.8)	191 (8.0)	55 (10.3)	31 (8.5)	55 (10.6)	121 (14.5)	

Continuous data are expressed as mean ± SD. Categorical data are expressed as number of cases (percent)

^a^Unknown data were not included in the statistical tests

^b^ *P* values were calculated using one-way ANOVA test (continuous variables) and Chi square or exact test (categorical variables)

### OS and CSS by stage and molecular subtype

The median follow-up time was 79.0 months, ranging from 0.3 to 119.9 months. Of the 4640 female breast cancer patients, 3976 (85.7%) were alive, and 664 (14.3%) died before December 31, 2015. The 5-year OS rate was 89.4% (95% confidence interval [CI] 88.5%–90.3%), and the 5-year CSS rate was 90.3% (95% CI 89.4%–91.1%) for all the patients. Both OS and CSS differed significantly by stage and molecular subtype (all *P* < 0.01).

**Fig. 1:**
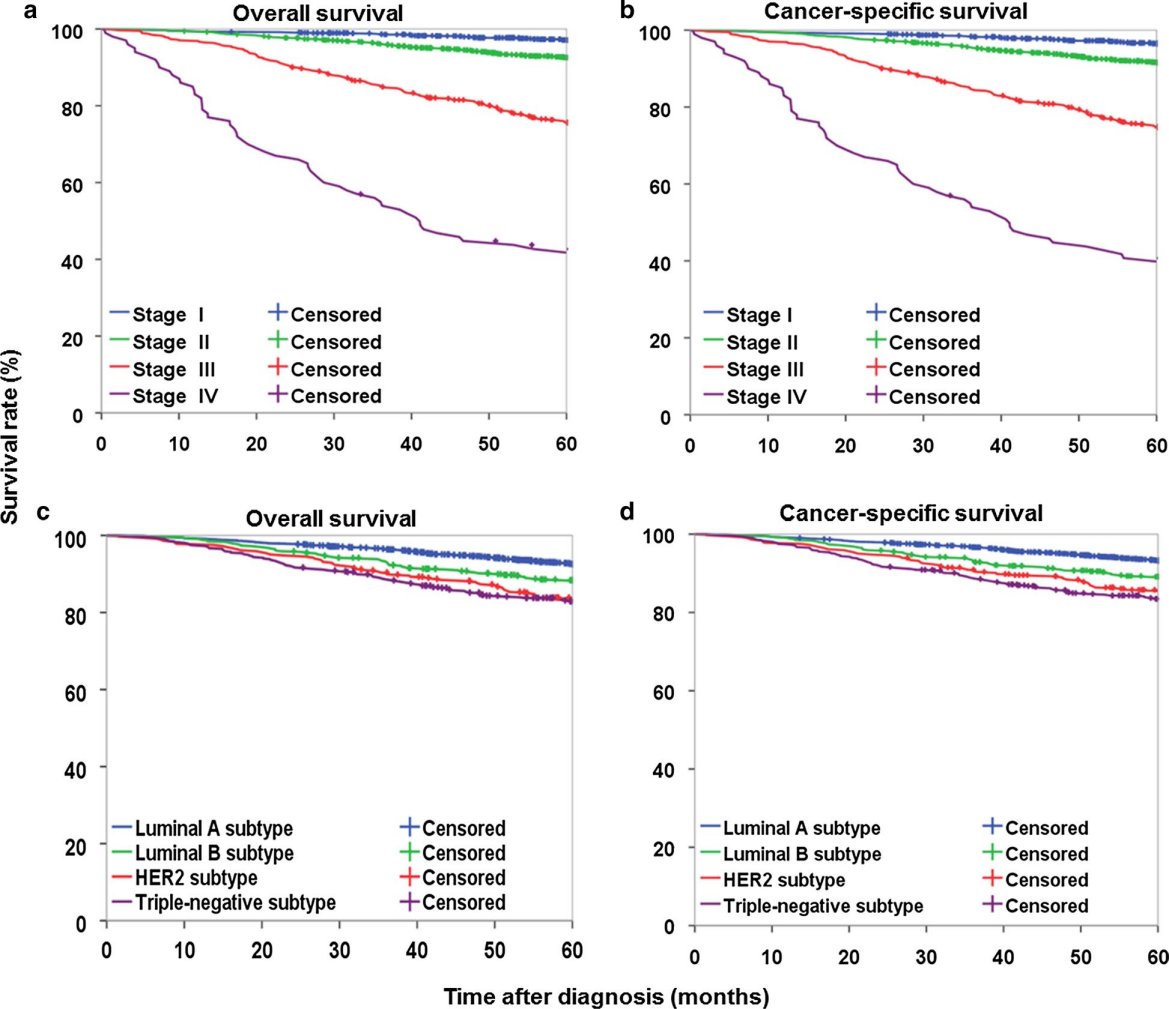
Survival curves of female patients with breast cancer at different stages at diagnosis or of different molecular subtypes. **a** Overall survival curves of patients according to tumor stage. **b** Cancer-specific survival curves of patients according to tumor stage. **c** Overall survival curves of patients according to molecular subtype. **d** Cancer-specific survival curves of patients according to molecular subtype. Log-rank test showed significant differences in survival among groups in the four panels (all *P* < 0.01)

For patients with stage I, II, III, and IV breast cancer, the 5-year OS rates were 96.5%, 91.6%, 74.8%, and 40.7%, respectively (Fig. 1a); the 5-year CSS rates were 97.1%, 92.6%, 75.6%, and 42.7%, respectively (Fig. 1b). Patients with late-stage disease had much lower 5-year survival rates than those with early-stage disease (Table 3).

**Table 3:** Subgroup survival analysis for patients with early- and late-stage breast cancer (%)

Characteristic	Early-stage					Late-stage				
	Total (cases)	5-year OS rate (%)	*P* value^b^	5-year CSS rate (%)	*P* value^b^	Total (cases)	5-year OS rate (%)	*P* value^b^	5-year CSS rate (%)	*P* value^b^
Histological grade			< 0.01		< 0.01			< 0.01		< 0.01
Well differentiated	283	96.8		97.5		25	92.0		92.0	
Moderately differentiated	1971	94.3		95.0		359	74.7		76.0	
Poorly differentiated	685	90.3		91.3		155	60.1		61.4	
Unknown^a^	575	92.7		94.0		134	63.2		63.2	
Histological type			0.66		0.41			0.89		0.73
Ductal	3089	93.4		94.1		597	70.1		71.2	
Lobular	103	92.1		95.0		26	63.9		63.9	
Others	322	94.1		95.6		50	67.9		67.9	
Molecular subtype			< 0.01		< 0.01			< 0.01		< 0.01
Luminal A	1897	95.2		95.8		299	77.1		77.8	
Luminal B	390	94.0		94.2		90	69.1		71.3	
HER2	256	90.9		92.4		78	58.3		61.7	
Triple-negative	371	88.6		89.4		91	60.4		60.4	
Unknown^a^	600	91.7		93.3		115	65.8		65.8	

Of the total 4640 patients, 453 with unknown stage at diagnosis were not included in the analysis

*OS* overall survival, *CSS* cancer-specific survival

^a^Unknown data were not included in the statistical tests

^b^ *P* values were calculated using log-rank test

For patients with luminal A, luminal B, HER2, and triple-negative subtypes of breast cancer, the 5-year OS rates were 92.6%, 88.4%, 83.6%, and 82.9%, respectively (Fig. 1c); the 5-year CSS rates were 93.2%, 89.1%, 85.4%, and 83.5%, respectively (Fig. 1d). Patients with HER2 and triple-negative subtype tumors had lower survival rate than those with luminal A and luminal B subtype tumors (Table 4).

**Table 4:** Survival of patients with breast cancer according to different stages and molecular subtypes

Characteristic	5-year OS rate [% (95% CI)]	5-year CSS rate [% (95% CI)]
Overall	89.4 (88.5–90.3)	90.3 (89.4–91.1)
Stage		
I	96.5 (95.4–97.4)	97.1 (96.1–97.9)
II	91.6 (90.3–92.7)	92.6 (91.4–93.6)
III	74.8 (71.0–78.2)	75.6 (71.8–78.9)
IV	40.7 (31.0–50.1)	42.7 (32.8–52.1)
Molecular subtype		
Luminal A	92.6 (91.5–93.6)	93.2 (92.1–94.2)
Luminal B	88.4 (85.3–90.9)	89.1 (86.1–91.5)
HER2	83.6 (79.3–87.0)	85.4 (81.3–88.7)
Triple-negative	82.9 (79.3–85.9)	83.5 (79.9–86.4)

*OS* overall survival, *CSS* cancer-specific survival, *CI* confidence interval

### Multivariate Cox regression analysis

Multivariate Cox regression analysis (Table 5) showed that age was an independent prognostic factor for OS and CSS. High BMI was associated with short OS, whereas no significant association was observed between BMI and CSS (*P* = 0.14). Patients with poorly differentiated tumor had shorter survival than those with well differentiated tumor. OS and CSS were significantly shorter among women with HER2 and triple-negative subtypes than those with luminal A subtype. Compared with patients at stage I, patients at stages II, III, and IV had significantly shorter OS and CSS.

**Table 5:** Multivariate analysis of overall survival and cancer-specific survival using Cox proportion hazards modeling

Characteristic	OS		CSS	
	HR^a^ (95% CI)	*P* value^a^	HR^a^ (95% CI)	*P* value^a^
Age	1.03 (1.02–1.05)	< 0.01	1.03 (1.01–1.04)	0.01
BMI	1.03 (1.01–1.06)	0.02	1.02 (0.99–1.05)	0.14
Menopausal status				
Premenopausal	1.00		1.00	
Postmenopausal	0.80 (0.59–1.09)	0.16	0.81 (0.58–1.12)	0.21
Histological grade				
Well differentiated	1.00		1.00	
Moderately differentiated	1.60 (0.94–2.71)	0.08	1.50 (0.87–2.59)	0.14
Poorly differentiated	2.14 (1.24–3.70)	0.01	2.02 (1.15–3.55)	0.02
Histological type				
Ductal	1.00		1.00	
Lobular	0.91 (0.33–2.46)	0.85	0.77 (0.24–2.44)	0.66
Others	0.94 (0.56–1.59)	0.83	0.85 (0.48–1.52)	0.59
Stage				
I	1.00		1.00	
II	2.19 (1.57–3.06)	< 0.01	2.24 (1.56–3.22)	<0.01
III	6.24 (4.40–8.87)	< 0.01	6.89 (4.72–10.05)	<0.01
IV	25.77 (16.36–40.59)	< 0.01	29.48 (18.33–47.44)	<0.01
Molecular subtype				
Luminal A	1.00		1.00	
Luminal B	1.26 (0.93–1.72)	0.14	1.31 (0.95–1.80)	0.10
HER2	1.50 (1.08–2.09)	0.02	1.47 (1.03–2.09)	0.03
Triple-negative	1.83 (1.37–2.43)	< 0.01	1.89 (1.39–2.55)	< 0.01

^a^HRs, 95% CIs, and *P* values were calculated using the adjusted multivariate Cox proportional hazard model

*OS* overall survival, *CSS* cancer-specific survival, *HR* hazard ratio, *CI* confidence interval, *BMI* body mass index

## Discussion

The present study demonstrated the influences of stage at diagnosis and molecular subtype on the survival of breast cancer patients with a large sample size. We found that survival varied significantly by stage at diagnosis and molecular subtype. Patients with late-stage disease had much lower survival rates than those with early-stage disease; patients with HER2 and triple-negative subtype tumors had lower survival rates than those with Luminal A or Luminal B subtype tumors.

The overall 5-year CSS rate in the present study was 90.3%, which was much higher than the estimates around China [16], while similar to those in developed countries. The relative survival estimates were 89.7% in the United States (2007–2013) [17] and 89.8% in Australia (2008–2012) [18]. As one of the largest cities and the capital of China, Beijing has more advanced medical facilities and health resources than most of other cities in China [19]. Patients diagnosed with breast cancer in Beijing could receive better supportive care and expect more favorable treatment outcome. These may all together contribute to the similar breast cancer patient survival rates between Beijing and developed countries.

Stage at diagnosis is a key prognostic factor of breast cancer [20, 21]. In western countries such as Canada, Denmark, Norway, Sweden, and the United Kingdom, about 30.1%–45.2% patients were diagnosed with stage I, 39.0%–47.7% with stage II, 3.5%–15.3% with stage III, and 2.9%–6.9% with stage IV diseases [20]. Our population showed a higher proportion of stage III and stage IV diseases compared with those in European countries such as Norway, Sweden, and the United Kingdom, which may plead for enforced screening program for primary prevention. Considering most of the hospitals involved in our study are tertiary-level medical institutions and a portion of stage IV breast cancer patients diagnosed at relatively low-level hospitals did not receive effective treatment or adopted conservative treatment, the proportion of patients with stage IV disease in the present study may be lower compared with the figure in actual population.

Compared with the reported survival rates in two periods in Beijing, 66.3% (1982–1983) and 74.2% (1987–1988) [22], the survival rate of breast cancer patients was remarkably increased in the present study. The improvement in treatment may contribute to the prolonged survival over the past decades [23, 24]. Besides, with the development of economy and society, citizens may have better awareness on health and early diagnosis of breast cancer. A better stage distribution between 2006 and 2010 compared with those in the past decades may also be an important contributor to the prolonged survival. People living in Beijing are more likely to have high socioeconomic status, to be literal on health, and to have access to good medical service to detect precancerous lesions compared with those in most other cities in China. Precancerous lesions in the breast have visible symptoms with favorable prognosis. An amount of women would go to the hospital when they found palpable masses in the breast. Besides, women in Beijing will easily have access to knowledge on cancer prevention which in turn enhances their awareness on self-exams. Clinical breast examinations, including mammography and ultrasound, are vital approaches to an early diagnosis of the disease. Since 2008, breast cancer screening was performed in Beijing for resident women aged 40–60 years. In addition, since 2011, a biennial breast screening program, free of charge, has been launched in Beijing for women aged 35–64 years [25]. Moreover, awareness campaigns have been carried out in communities in turn to inform and educate people on the disease. It is expected that the survival of breast cancer patients in Beijing would be further prolonged in the future. Breast cancer screening programs have been implemented in developed countries for years [26, 27]. The United States Prevention Services Task Force (USPSTF) recommended biennial mammography screening for women aged 50–74 years and selective screening for those aged 40–49 years [27]. The international agency for research on cancer (IARC) have concluded that breast cancer mortality is generally reduced by screening especially for women aged 50–69 years [28]. However, there is no nationwide breast cancer screening program in China. Chinese women tend to have dense breasts [29], which have low sensitivity and specificity in mammography screening [30]. Ultrasound is a better choice for screening and showed higher efficacy on identifying early-stage patients than mammography from high-risk Chinese women [31]. Moreover, the uneven distribution of health care resources and services make it difficult to carry out uniform screening method in China. Spatio-temporal analysis revealed the cluster pattern for the incidence of female breast cancer, which was useful for a better allocation of health care resources [32]. Large randomized controlled trials of population-based studies are in urgent need among the Chinese population.

Breast cancer is a heterogeneous disease with different molecular subtypes. Our study enabled us to further examine the subtypes of the disease and their associations with survival. The proportion of HER2 subtype in the Chinese population is relatively higher than that for whites in the US, and the proportion of triple-negative subtype is lower than that for African Americans [33], which is consistent with the published findings that the proportion of HER2 subtype was higher in Asian and the triple-negative subtype was more frequent in Black [34].

Significant survival disparities were observed between hormone receptor-positive tumors (luminal A and luminal B) and hormone receptor-negative tumors (triple-negative and HER2). Breast cancer is a hormone-dependent cancer. ER-positive or PR-positive tumors account for the majority of breast cancers diagnosed. The survival differences among patients with various molecular subtypes of breast cancer reflected the distinct treatment responses associated with different expression statuses of hormone receptors. Tamoxifen and aromatase inhibitors are routine drugs for hormone receptor-positive tumors, and patients with HER2-overexpressed tumors can be treated with trastuzumab [35–37]. Triple-negative tumors can only be treated with surgery, radiation therapy, and/or chemotherapy [38]. Survival of patients with HER2 subtype disease was similar to that of patients with triple-negative subtype disease in the present study. The reason may be due to the insufficient use of targeted drugs for patients with HER2-deficient tumors [39]. The targeted drugs can be a large financial burden for the patients and are not covered by the basic medical insurances for urban residents and the new-rural cooperative medical system insurance in Beijing. Furthermore, not all HER2-positive breast cancer patients can benefit from trastuzumab therapy [40]. Therefore, the improving health insurance coverage may be needed. More advanced therapies and health care policies on HER2 and triple-negative molecular subtypes are of great importance.

BMI and histological grade are significant indicators associated with breast cancer patient survival apart from stage at diagnosis and molecular subtype, which had been reported in previous studies [41, 42]. Application of BMI and histological grade in the clinical practice can help clinicians predict the prognosis of breast cancer patients. Besides, relative studies showed that early age at menarche, late age at menopause, nulliparity, late age at first birth, and limited breastfeeding were also associated with increased risk of breast cancer in Chinese [43]. Activities on promoting the awareness of risk factors in women may improve the cognition of breast cancer, which could help women in risk pay more attention on cancer detection or recognize the disease in early stage.

The Beijing cancer registry was established in 1976, which has a long history and good working basis on cancer incidence and mortality registration in China. Considering some breast cancer patients were lack of effective treatment in the population, the 5-year CSS rate in Beijing in the present study may be higher than that of population-based estimates. However, the multi-center nature of the study enables an inclusion of about 40% of the total patient population, which leads to a fair degree of external generalizability of the survival estimates of patients with breast cancer at different stages and of different molecular subtypes. Hospital-based cancer registration can provide extensive, detailed, and reliable information on clinical characteristics. As hormone receptor status and cancer stage are not routinely required by registries, limited high-resolution survival researches in China hindered the cognition of breast cancer and the development of its treatment. This study can help explore hospital-based cancer registration pattern, assess data accessibility, and provide basic information for evidence-based medicine.

## Conclusions

The present study demonstrated the influences of stage at diagnosis and molecular subtype on the survival of breast cancer patients with a large sample size. Stage at diagnosis and molecular subtype are independent prognostic factors associated with breast cancer patient survival. Patients with late-stage diseases had much lower survival rates than those with early-stage diseases, and patients with HER2 or triple-negative subtype tumors had lower survival rates than those with luminal A or luminal B subtype tumors. Cancer screening is encouraged for the early detection and early diagnosis of breast cancer. More advanced therapies and health care policies are needed on HER2 and triple-negative subtypes. Our study can also provide basic information for clinicians and policy-makers in further actions on breast cancer treatment research and health care and serve as a baseline for the establishment of hospital-based cancer registration system.

## Abbreviations

OS: overall survival
CSS: cancer-specific survival
BMI: body mass index
ER: estrogen receptor
PR: progesterone receptor
HER2: human epidermal growth factor 2
FISH: fluorescence in situ hybridization
HR: hazard ratio
CI: confidence interval
USPSTF: US Prevention Services Task Force
IARC: International Agency for Research on Cancer
ANOVA: analysis of variance
SD: standard deviation
